# Self-Rated Health Status as a Predictor of Executive Function in Older Latinos

**DOI:** 10.1093/geroni/igaa057.1063

**Published:** 2020-12-16

**Authors:** Jacqueline Guzman, Yuliana Soto, David Marquez, Susan Aguinaga

**Affiliations:** 1 University of Illinois at Urbana-Champaign, Urbana, Illinois, United States; 2 University of Illinois–Urbana-Champaign, champaign, Illinois, United States; 3 University of Illinois at Chicago, Chicago, Illinois, United States

## Abstract

Latinos have high risk of Alzheimer’s disease and related dementias (ADRD). Self-rated health (SRH) has been used to predict cognitive decline. Early detection of executive function changes may help identify those at higher risk of cognitive decline. The purpose of this study was to examine the relationship between SRH and executive function in Latinos. Latinos (N=333, 84.4% female, Mage= 64.9 ± 7.08) from the BAILA randomized controlled trial self-rated their health as 1) poor/fair, 2) good, and 3) very good/excellent. Executive function was assessed by the Trail-making B, Verbal Fluency, Stroop C & CW, and the Digit Modality tests and stratified by SRH. One-way analysis of variance showed that the effect of SRH was significant for Trails B, F(2,298)=4.01, p=.019 and Stroop CW, F(2,298)=3.07, p=.048. Tukey’s test indicated that participants who rated their health as fair/poor took longer to complete Trails B (M=196.78±83.0 seconds) compared to those who rated their health as good (M=185.25 ± 85.1 seconds) and very good/excellent (M=149.25±95.3 seconds). Stroop CW results demonstrated that those in the fair/poor health category scored lower (M=17.22±6.6) than those in good (M=19.70±8.5 words/minutes) and very good/excellent health categories (M=18.73±8.2 words/minute). In sum, the results suggest SRH is related to executive function such that lower categories of SRH are indicative of poorer executive function. SRH might be used as a proxy for executive function and as a tool that community leaders can use to identify individuals at high risk of ADRD in need of behavioral interventions.

